# A Wearable Context-Aware ECG Monitoring System Integrated with Built-in Kinematic Sensors of the Smartphone

**DOI:** 10.3390/s150511465

**Published:** 2015-05-19

**Authors:** Fen Miao, Yayu Cheng, Yi He, Qingyun He, Ye Li

**Affiliations:** 1 Key Laboratory for Health Informatics of the Chinese Academy of Sciences (HICAS), Shenzhen Institutes of Advanced Technology, 1068 Xueyuan Boulevard, Shenzhen 518055, China; E-Mails: fen.miao@siat.ac.cn (F.M.); yy.cheng@blestech.com (Y.C.); qy.he@siat.ac.cn (Q.H.); 2 Shenzhen College of Advanced Technology, University of Chinese Academy of Sciences, Shenzhen 518055, China; 3 High-field Magnetic Resonance Department, Max Planck Institute for Biological Cybernetics, Tuebingen 72076, Germany; E-Mail: yi.he@tuebingen.mpg.de

**Keywords:** context-aware, power control, physical activity recognition, wearable ECG sensor

## Abstract

Continuously monitoring the ECG signals over hours combined with activity status is very important for preventing cardiovascular diseases. A traditional ECG holter is often inconvenient to carry because it has many electrodes attached to the chest and because it is heavy. This work proposes a wearable, low power context-aware ECG monitoring system integrated built-in kinetic sensors of the smartphone with a self-designed ECG sensor. The wearable ECG sensor is comprised of a fully integrated analog front-end (AFE), a commercial micro control unit (MCU), a secure digital (SD) card, and a Bluetooth module. The whole sensor is very small with a size of only 58 × 50 × 10 mm for wearable monitoring application due to the AFE design, and the total power dissipation in a full round of ECG acquisition is only 12.5 mW. With the help of built-in kinetic sensors of the smartphone, the proposed system can compute and recognize user’s physical activity, and thus provide context-aware information for the continuous ECG monitoring. The experimental results demonstrated the performance of proposed system in improving diagnosis accuracy for arrhythmias and identifying the most common abnormal ECG patterns in different activities. In conclusion, we provide a wearable, accurate and energy-efficient system for long-term and context-aware ECG monitoring without any extra cost on kinetic sensor design but with the help of the widespread smartphone.

## 1. Introduction

Cardiovascular diseases are the principle causes of death worldwide [1,2]. The continuous electrocardiogram (ECG), which indicates the overall rhythm of the heart and can be monitored using non-invasive electrodes on the chest or limbs, has been demonstrated with prognostic significance for cardiovascular diseases [3]. Therefore, more than 24 h or 48 h ambulatory ECG monitoring is becoming more and more important in both homecare and clinical settings to prevent cardiovascular disease and detect symptomatic signs for patients with uncommon events [4]. In another aspect, as the characteristics of ECG signals are highly dependent on the user’s physical status, a combination method to monitor ECG and physical activity is in great need [5]. Combining with context information from activity status is beneficial to improve diagnosis accuracy on the ECG signals, identify the most common abnormal ECG patterns in different activities, and evaluate the cardiac functions for the clinicians.

Usually, patients have to carry a bulky instrument for continuous ECG monitoring, which restricts their mobility and makes them uncomfortable with so many electrodes and cables around their bodies. There is a growing demand for small-size, compact wearable ECG acquisition system [6,7]. With the development of electronics over the past several years, more and more cost-effective approaches have been proposed to replace conventional methods for ambulatory ECG monitoring [8,9,10]. In 2012, Christian *et al*., introduced a system-level low-power wireless sensor for long-term biomedical signal monitoring. The proposed solution of an ECG circuits was demonstrated to lead to 2.5 times power saving [8]. In 2014, a mixed-signal system-on-chip (SoC) integrated analog font-end (AFE) with DSP back-end was proposed in [9]. The wireless ECG monitoring system comprised of the proposed SoC and Bluetooth protocol can implement a power consumption of 13.34 mW while transmitting ECG signal with 256 Hz sampling rate via Bluetooth. There are also some portable commercial devices until now, such as eMotion from MEGA Electronics Ltd [11] and wireless health monitoring system from IMEC [12], both of them are equipped with a Bluetooth module. Even though the reported ECG signal acquisition circuits are outstanding in architecture or power optimization, they do not work as a completed system to provide a lot of medical functions such as arrhythmias detection and context-aware information in real-life application.

In another aspect, the widespread of smartphone with powerful computing capability and high-speed data access via wifi, 3G/4G cellular network, makes the ECG smartphone applications available [13,14,15]. With high-resolution touch-screen and universal communication interfaces, the smartphone based system can not only exhibit real-time ECG signals sent from the ECG sensor, but also transmit it to remote computing server to provide healthcare service [15]. Moreover, the smartphone can be used to recognize physical activity based on built-in sensors [16,17] with high accuracy and reliability. Therefore, how to take advantage of available device to reduce the power consumption of the ECG monitoring system while providing useful diagnosis information for the users is necessary.

In this work, we describe a wearable context-aware ECG monitoring system comprised of a self-designed integrated ECG sensor for continuous, long-term remote ECG monitoring and a smartphone for abnormal ECG patterns and physical activity recognition. The ECG acquisition sensor implemented with a full custom, fully integrated and low power AFE is presented in our study to minimize the size and power consumption. With Bluetooth technology, the acquired ECG signal is sent to user’s smartphone for real-time display and arrhythmias identification. Meanwhile, combining with the context information provided by the built-in kinematic sensors (triaccelerometer, gyroscope, and magnetic sensor) in the smartphone, this system can recognize a user’s physical activities and thus improve the accuracy for identifying ECG abnormal patterns. Compared with previous studies, we provide a platform for low-power, long-term and accurate ECG monitoring with a self-designed ECG sensor, activity recognition and then data fusion for improving the diagnosis accuracy without any extra cost on kinetic sensor design but with the help of the widespread smartphone.

The remainder of the paper is organized as follows. In Section 2, we present an overview of the proposed context-aware ECG monitoring system, a combination of wearable ECG signal acquisition sensor and three built-in kinematic sensors in the smartphone for identifying physical activity. In addition, the software on the smartphone for data fusion and analysis is introduced in Section 2. Then in Section 3, the experimental results for identifying physical activities and the effectiveness of improving diagnosis accuracy while combining with physical activity are given. We conclude our study in Section 4, with an outlook for further research.

## 2. The Proposed Context-Aware ECG Monitoring System

### 2.1. System Architecture

**Figure 1:**
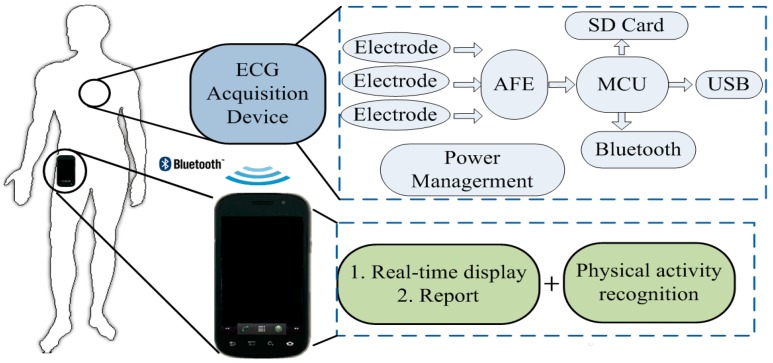
Schematic diagram of proposed context-aware electrocardiogram (ECG) monitoring system.

The block diagram of the proposed ECG monitoring system combined a wearable ECG acquisition sensor with a smartphone is described in Figure 1. The ECG sensor was developed following YY1139-2000 standard, a pharmaceutical industry standard of China for single and multichannel electrodigraph, which is evolved from EC13 national standard but more conform to local situation. In the ECG acquisition sensor, signal is amplified and filtered by a single chip of AFE module, then in MCU module the analog signal from AFE is converted to digital signal. After processed with compression algorithm, the digital signal is recorded in SD card or transmitted to smartphone for real-time display. Meanwhile, a USB port is equipped in the device for transmitting the signals which have been saved in the SD card to personal computers and then to the cloud platform for further analysis.

The ECG signals transmitted to smartphone are real-time displayed on screen, with a brief report provided from the automatic analysis approach in the software or professional advices provided from the remote server. The built-in kinematic sensors of the smartphone are used to recognize the individual’s physical activity and thus help to improve the diagnosis accuracy for detecting abnormal patterns.

### 2.2. Architecture of Wearable ECG Sensor

**Figure 2:**
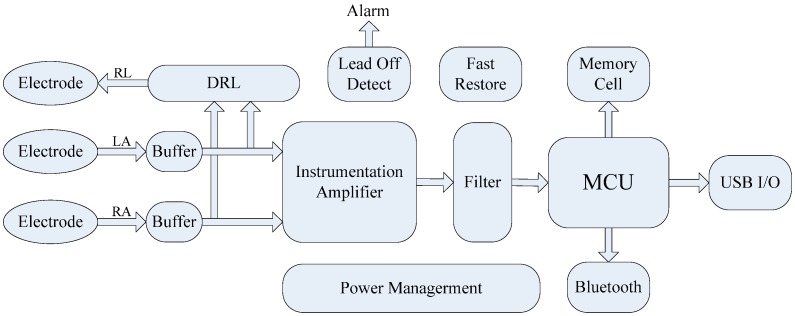
Architecture of traditional acquisition device.

The block diagram of traditional implementation of ECG acquisition device is presented in Figure 2, in which the circuit consists of a traditional instrument amplifier and Sallen-Key or Nyquist low pass filter [18], and some external function circuits for realistic ECG detection. The system also needs a lot of discrete components which occupy a large area that hindering the aim for wearable and low power design.

The architecture of the proposed wearable sensor is shown in Figure 2. In the module of full custom, fully integrated, low power AFE, we integrated all the function circuits in traditional implementation presented in Figure 2. The integrated AFE in our study consists of an input/output buffer, full differential amplifier (DA) with high pass function, second Gm-C low pass filter, additional amplifying stage, DRL circuit, lead-off detecting circuit, fast restore function, and a power management module to provide a stable working voltage and current.

### 2.3. Full Custom, Full Integrated AFE

**Figure 3:**
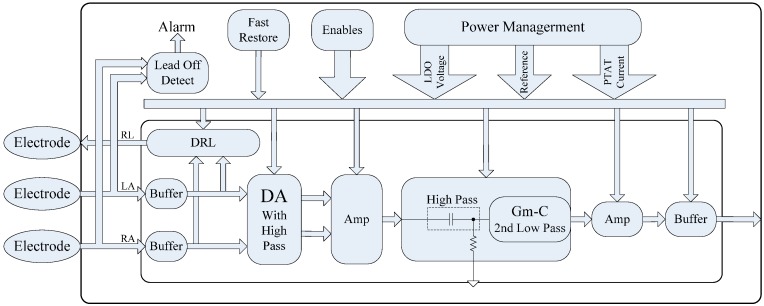
Block diagram of the analog front-end (AFE).

The detailed block diagram of the proposed AFE is shown in Figure 3. In place of a separate analog-to-digital convert module, the inherent analog-to-digital function in the MCU module was used in the proposed system to improve efficiency.

In order to resolve the challenge of tradeoff between size and bandwidth of the circuit, we designed a full DA module with high pass function using pseudo-resistor technology [19] and a second low pass Gm-C filter [20,21], instead of traditional instrumentation amplifier and Sallen-Key or Nyquist low pass filter. The designed module is able to realize a very low pass band on the chip. At the same time, the DA module using the capacitance amplifying structure can also eliminate the DC offset voltage of electrodes which limit the gain of the first stage amplifier.

In addition, because of the equivalent resistance of human body, combined with the principle that equivalent input resistance of the AFE should be larger than 10 MΩ to eliminate the effect of voltage divider, we need an input buffer to get a high enough input resistance and depart the electrode from amplifying stage in case of some other environmental interference factors. A passive high pass filter is proposed to ensure the DC working voltage with a cutoff frequency of 0.5 Hz. A second low pass Gm-C filter is integrated and followed by an additional amplifying stage to get a higher gain. We integrate a traditional right-leg driven circuit to improve the common mode rejection ratio (CMRR) performance. In order to realize the disconnection between electrode and human skin for a wearable, comfortable device for long-term monitoring, we design a lead off circuit with equivalent resistance larger than 10 MΩ. In addition, a fast restore function circuit is integrated to help AFE get back to working state fast from abnormal state. A power management module is designed to provide a stable working voltage and current.

### 2.4. Scheduling of MCU

**Figure 4:**
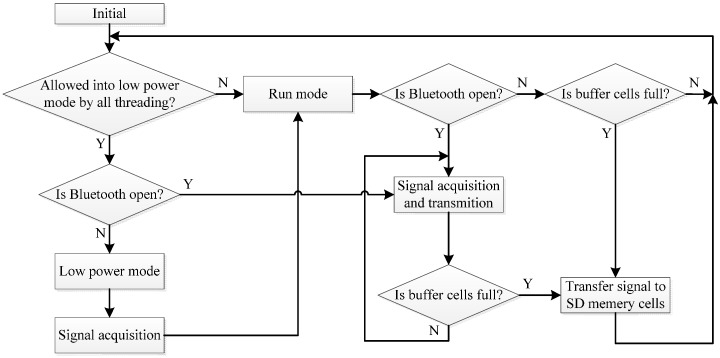
Flow diagram of the scheduling of a micro control unit (MCU).

We utilize the analog-to-digital (AD) function embedded in the MCU module to convert the analog signal from AFE to digital signal for efficiency. In pursuit of low power requirement, the device can work in turn between two modes in scheduling of MCU, that is, normal run mode and low power mode. In the low power mode, the signal acquired is saved to buffer memory cells at regular time, when the storage space of buffer cells is full, an interrupt signal will be generated and make the device to work in normal run mode. In the normal run mode, central processing units start to work and transfer the signal saved in buffer cells to secure digital memory card or transmit the signal to smartphone. The process of the signal transferring only takes decades of milliseconds, so in most of time the device works in low power mode. The block diagram of main scheduling of MCU is described in Figure 4.

### 2.5. Physical Activity Recognition with Built-in Kinetic Sensors in the Smartphone

As presented above, smartphone can be used to detect the fall state [22,23,24] and recognize physical activities with a degree of accuracy comparable to that of the tri-axial accelerometer. In our previous study [25], we utilized a smartphone to recognize 5-type physical activity with three built-in kinematic sensors (tri-accelerometer, gyroscope, and magnetic sensor), including static, walking, running, going upstairs and going downstairs. The experiment results on 8097 activity data have demonstrated the proposed approach can realize a high degree of accuracy with recognition rate of 89.6%. We also evaluated the impact of different pockets and orientations for activity recognition in [25], the experimental results demonstrated that the proposed solution is insensitive to four orientations including head upward and face inward, head upward and face outward, head downward and face inward, head downward and face outward. For the six different pockets, from the results presented in Figure 9b in [25], the features from trouser pockets are with higher variability compared with coat pockets, as bigger acceleration is produced from the lower limbs than that from the trunk part while the subject is moving. Moreover, the features from different trousers pockets are similar. We also evaluated the standard derivation of the mean acceleration data among the three activities including rest, walking and running for each pocket presented in [25], the results showed that the standard derivations are 4.87 (with mean acceleration data of 0.85 for rest, 3.24 for walking and 10.22 for running) and 5.06 (with mean acceleration data of 0.72 for rest, 3.41 for walking and 10.53 for running) for right rear trousers pocket and right front trousers pocket separately, while 3.50 for left coat pocket (with mean acceleration data of 1.08 for rest, 3.51 for walking and 7.99 for running). In another word, the acceleration data from trousers pockets are with higher discrimination than coat pocket. Therefore, the similar solution will be used in this study to recognize typical types of physical status that have significant influence on the ECG pattern, including rest, walking and running, with the smartphone in any trousers pockets. The running and walking status was distinguished based on the ambulation speed, which was defined in [26]. Accordingly, the speed below 84 m/min is defined as walking, otherwise it is marked as running.

**Figure 5:**
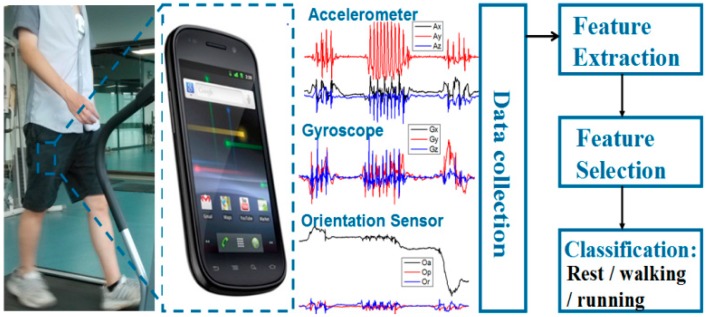
The block diagram of the physical activity recognition scheme.

As shown in Figure 5, the built-in accelerometer, gyroscope, and the orientation sensor of a smartphone which reflected acceleration, angular velocity, and orientation of physical activity were used to collect the information for classification. In order to reduce the influence of measurement noise, we used a low-pass filter with 10 Hz cutoff frequency to pre-process the data prior to the activity classification. In addition, the sliding window approach was employed to reduce the bias arisen from sensor sensibility and noise by segmenting the signals into multiple small overlapped windows. At a sampling frequency of 25 Hz, each window with 50% overlap represents 1.6 s. Five signals, including the signal magnitudes of the accelerometer and gyroscope sensor, the X, Y, Z direction value of the magnetic sensor, were collected for each sample. Then 30 features (mean, standard deviation, median, skewness, Kurtosis, Inter-quartile-range of the five signals for each window) were extracted for classification. After that, we classified activities (rest, walking and running) from daily life data employing decision trees in WEKA environment [27] using the extracted 30 features. The decision tree was created using the J48 algorithm, an open source Java implementation of the C4.5 decision tree algorithm in the WEKA tool. It is implemented in the following ways: (1) At each node of the tree, the attribute that splits the dataset into subsets concentrated in one class or the other most effectively, that is, with the highest normalized information gain, is chosen to make a decision; (2) To avoid the over-fitting problem, a stop-splitting rule is required to control the growth of the tree. In our study, the stop-splitting rule was defined that the minimum number of objects at each terminal node should be larger than 30. The J48 is implemented on the basis that if a smaller tree structure is with comparable performance to a larger one, the smaller one would be chosen.

### 2.6. Software on Smartphone

The software on the smartphone was developed at Android operating system. It mainly consists of a feature extraction program using weighted average beat subtraction (WABS) method and maximum likelihood estimation (MLE) method [28], a physical activity recognition program, a Bluetooth receiving program, a display program and interactive interface program. Feature extraction program extracts features of ECG signal from single lead ECG recordings and according these features to identify 10-type arrhythmias. Physical activity recognition program acquires the information from built-in accelerometer, gyroscope and orientation sensors, and then extracts features from the information, which were utilized to recognize three typical activities (rest, walking and running). Bluetooth receiving program is in charge of the connection between ECG sensor and smartphone. Display program unpacks the received ECG data and obtains the result from physical activity recognition. The software interface of smartphone terminal will be introduced in Results and Discussion part.

A smartphone (Samsung, I9100GALAXYSII, 125.3 × 66.1 × 8.49 mm, 116 g, Android OS 2.3) was used to carry out the experiments in our study. The smartphone is equipped with a built-in triaxial accelerometer (STM K3DH) with 19.6 m/s^2^ maximum range and 0.019 m/s^2^ resolution, a triaxial gyroscope sensor (STM K3G) with 34.9 rad/s maximum range and 0.0012 rad/s resolution, and a triaxial magnetic field sensor (Asahi Kasei AK8973) with 2000 μT maximum range and 0.0625 μT resolution. We have tested the performance of the developed app with the smartphone. The test results showed that the CPU occupation is about 25% and battery usage is about 1mAh in one minute while running the APP in background. With a 1650 mAh battery capacity, the smartphone can provide more than 24 h activity recognition without other applications. The developed APP performs very well while running other applications including phone call, address book searching and camera.

## 3. Results and Discussion

### 3.1. Experimental Results of Proposed AFE

**Figure 6:**
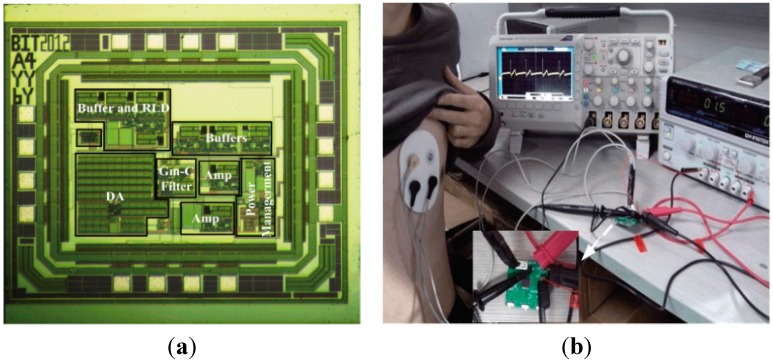
(**a**) The micro photograph; (**b**) Experiment of proposed AFE in ECG Acquisition device.

The photograph of proposed AFE with 0.18 μm CMOS technology SMIC process is presented in Figure 6a, the chip size is 1.3 mm × 1.1 mm and the core circuit only occupies 0.9 mm × 0.8 mm. Figure 6b shows the experimental platform to test and verify our AFE on human body, the testing board of our chip is also shown on the left-down corner, in which only a few discrete components are needed for measurement. The ECG was measured with a gain setting of 360 and the filter cutoff set of 0.5 ~ 120 Hz. The screenshot of the oscilloscope shows that different characteristics of the ECG are clearly visible and thus demonstrate that the AFE can function as well as expected. It can be used to measure signals in high quality while applied in ECG acquisition system.

### 3.2. Performance of Proposed ECG Acquisition Sensor

**Figure 7:**
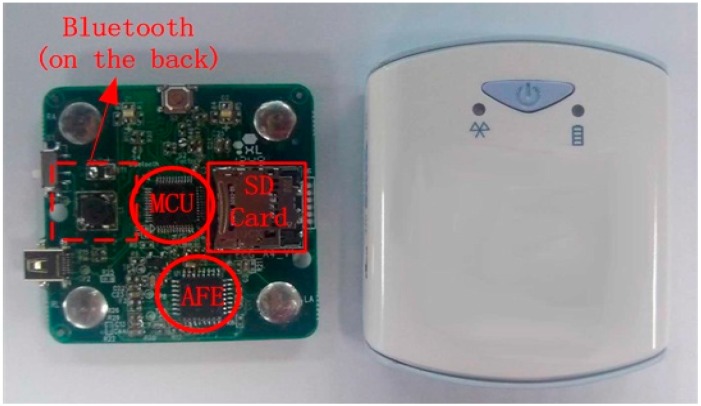
Photograph of proposed ECG Acquisition device.

Figure 7 shows the photograph of proposed acquisition sensor at the left and its appearance of final package. The sensor is very small for wearable monitoring application with a size of only 58 × 50 × 10 mm and a weight of 20 g.

**Figure 8:**
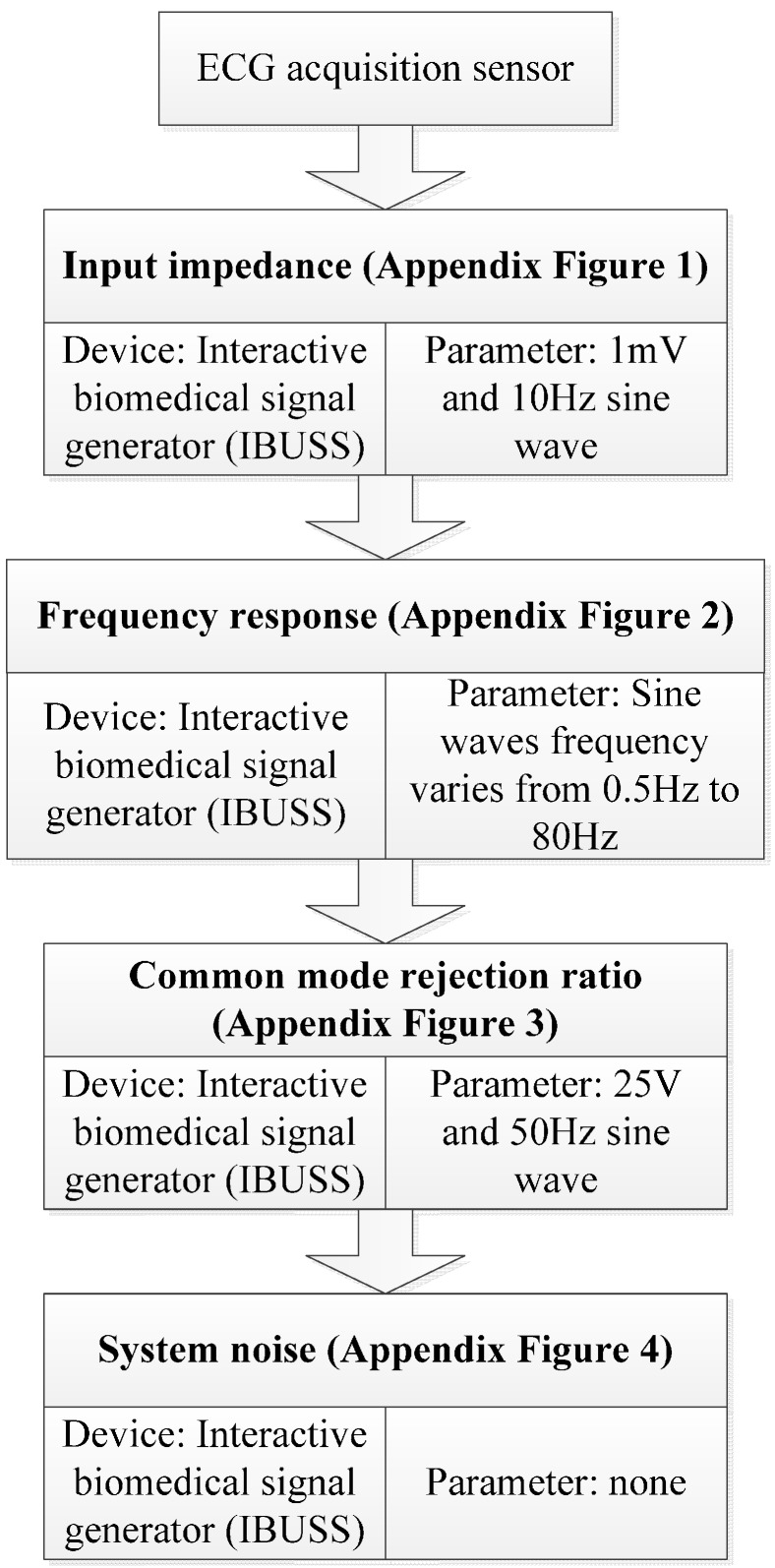
ECG acquisition sensor hardware validation procedure.

To validate the hardware performance of the proposed ECG sensor, we evaluated four main indexes including input impedance, frequency response, anti-jaming capability and system noise. The test procedure and corresponding device and setting parameters for all the indexes can be found in Figure 8. Table 1 gives a summary of the performance. The testbed for input impedance is presented in appendix Figure 1. The skew rate of 1% has demonstrated the performance in input impedance with the input impedance larger than 5 MΩ, and thus is in accordance with YY1139-2000. Appendix Figure 2 gives the testbed for frequency response. The sine waves with frequency varying from 0.5 Hz to 80Hz were inputted sequentially to verify the performance of frequency response. We evaluated the antijaming capability in terms of CMRR, which can be found in appendix Figure 3. The screen height of 4.2 mm demonstrated the CMRR was larger than 85 dB, and thus complied with YY1139-2000 perfectly. The testbed for system noise is presented in appendix Figure 4, a screen height of 0.4 mm showed the system noise was less than 15 μV, and also adhered to the rule in YY1139-2000.

**Table 1:** Performance summary of the ECG sensor hardware.

**Technology**	CMOS 0.18 μm
**Supply Voltage**	3 V
**Chip Size**	1.3 mm × 1.1 mm
**Input Impedance**	>5 MΩ
**Frequency Response**	<20 Hz for 8 inputted frequency
**CMRR**	>85 dB
**System Noise**	<15 μV
**Gain**	360
**Sampling Rate**	150 Hz, 250 Hz, 500 Hz
**Data Bit-Width**	32 bit

As the power consumption is a very important parameter for long-term continuously monitoring, we evaluated the total power consumption including AFE, MCU, SD card and other modules. The testbed for computing the power is demonstrated in Figure 6b, in which a DC power supply (GPS-2303C) is used to generate the voltage and an oscilloscope to exhibit the output current. The power is computed by product of the voltage and the current under the voltage. The power consumption computation was performed on each module separately and then the total device from a 3 V supply. For example, with the power supply of 3 V, this AFE consumes around 100 µA typically and its shutdown current is less than 1 µA, and thus the power consumption is 0.3 mW. For the long term monitoring or home-based application scenario, we don’t need to view the real-time signal but transmit the data once a day. So the wireless module is off at most time and the power consumption can be ignored while discussing the total power dissipation of the system.

Table 2 summarizes the power consumption of the proposed acquisition device and each module separately. It can be seen that the presented device only consumes around 12.5 mW total power dissipation, and the proposed AFE only consumes 2.4% of total power consumption. The main part of power consumption focus on the SD card, due to the writing process. However, the process of writing the acquired ECG data to SD card only takes tens of milliseconds, in most of time it is inactive.

**Table 2:** Power consumption of the proposed ECG acquisition device.

	AFE	MCU	SD Card	Power Module and Other Circuits
**Power Dissipation (mW)**	0.3	2.67	6 (on average)	3.53
**Power Dissipation (%)**	2.4	21.36	48	28.24
**Total Power (mW)**	12.5			
**Device Lifetime**	30 h (one 130 mAh AA battery)			

Table 3 summarizes the comparison of this work with other recent similar works and some commercial products in the market. In which the power consumption of other works are evaluated based on their data sheets. It can be seen that this work performs better than other works in those proposed aspects. In the future, the performance of higher CMRR, lower power consumption and lower noise, lower weight is our objective.

To validate the reliability of the proposed sensor on acquiring ECG data, we evaluated the sensor following the procedures presented in [29]. The validation protocol is comprised of two phases: The first phase is a laboratory test for evaluating the performance of the proposed sensor using an ECG signal generator; the second phase is a real-life experiment on subjects while performing a standard procedure including sitting, sit-to-stand, standing, stand-to-sit using the BIOPAC MP150 multi-channel physiological instrument.

**Table 3:** Performance comparison between our system and other recent similar works.

	This Work	Sensors 2013[30]	ISITME 2011	IEEE EMBC 2006 [31]	Holter ECG System (TLC4000) [32]	Holter Recording (DMS3004A) [33]
**Channel**	1	1	1	3	12	12
**Size**	5.8 × 5.0 × 1.0 cm^3^	5.8 × 5.0 × 0.4 cm^3^ (Without Package)	5.5 × 3.4 × 1.6 cm^3^ (Without Package)	N/A	N/A	8.8 × 5.5 × 2.1 cm^3^
**Supply (V)**	3	3	3.3	3	3	1.5
**Power (mW)**	12.5	84.83	115	375	312.5	25
**Storage**	SD Card	SD Card	N	SD Card	Build-in memory	Build-in memory
**Weight (g)**	20	38 (exclude battery)	20.7 (exclude battery)	N/A	N/A	100

In the first phase, simulated ECG signals with six rates varying from 30 to 200 BPM and four amplitudes from 0.15 to 1 mV were generated by the ECG generator. Each signal was recorded firstly by attaching the BIOPAC to the ECG generator to acquire 50 ECG waveforms for each amplitude and frequency setting, then the same measurements were repeated using the proposed sensor. The testbed is presented in appendix Figure 5a. We compared the two sets of signals in terms of cross-correlation coefficient of the signal and the QRS amplitude ratio, which can be seen in Table 4 and Table 5. From Table 4 and Table 5 we can see, the signals acquired from the proposed sensor are almost identical to those generated from the ECG generator. In the second phase, seven healthy subjects (five males and two females) were recruited to perform the following typical daily activities presented in Table 6 sequentially for 3 times. The baseline characteristics of the experimental subjects are: age 24.4 ± 6.05 years, height 173.7 ± 5.3 cm, and body mass index 21.1 ± 2.3 kg/m^2^. BIOPAC and the proposed sensor were used to collect the ECG signals simultaneously and the testbed is presented in appendix Figure 5b. We compared the acquired ECG signals between the BIOPAC and the proposed sensor in terms of QRS detection error, QRS amplitude ratio, QRS cross correlation and QRS detection delay, which were defined in [29]. The test results are presented in Table 7. From Table 7 we can see, the signals acquired from the proposed sensor are closely correlated to those collected from standard device in real-life settings.

**Table 4:** Average signal cross correlation between ECG generator device and the proposed sensor.

	Frequency (BPM)	30	60	80	120	160
Amplitudes (mV)						
**0.15**		0.985	0.983	0.983	0.984	0.985
**0.3**		0.983	0.985	0.984	0.984	0.983
**0.5**		0.984	0.983	0.983	0.986	0.983
**1**		0.983	0.984	0.983	0.984	0.985

**Table 5:** Average QRS amplitude ratio between ECG generator device and the proposed sensor.

	Frequency (BPM)	30	60	80	120	160
Amplitudes (mV)						
**0.15**		1	1	1	1	1
**0.3**		1	1	1	1	1
**0.5**		1	1	1	1	1
**1**		1	1	1	1	1

**Table 6:** Daily life activity.

Activity	Time Duration
Sitting	30 s
Sit-to-Stand	5 s
Standing	30 s
Stand-to-Sit	5 s

**Table 7:** Performance comparison between the proposed sensor and BIOPAC in real-life setting.

	Performance	QRS Detection Error No. (Avg ± std)	QRS Amplitude Ratio (Avg ± std)	QRS Cross Correlation (Avg ± std)	QRS Detection Delay No. (Avg ± std)
Activity					
Sitting		0.825 ± 0.836	0.987 ± 0.154	0.928 ± 0.137	0.687 ± 0.704
Sit-to-Stand		0.259 ± 0.117	1.082 ± 0.057	0.896± 0.106	0.524 ± 0.442
Standing		0.793 ± 0.689	1.061 ± 0.072	0.908 ± 0.174	0.712 ± 0.812
Stand-to-Sit		0.296 ± 0.124	0.899 ± 0.043	0.886 ± 0.157	0.465 ± 0.385

### 3.3. Physical Activity Recognition Result

In order to demonstrate the effectiveness of the proposed system, the recruited seven healthy subjects (five males and two females) were asked to perform the three activities (rest, walking and running) wearing the proposed ECG sensor on their chest and a smartphone in their any trousers pockets, as presented in Figure 5. We first evaluate the performance of physical activity recognition by smartphone’s built-in sensors for everyday activities. The subjects were asked to perform the activities in their own style and in a random order for overall 10 min. The speed was set to 4 km/h for walking and 8 km/h for running with a treadmill. All the activity data collected from the seven subjects were mingled together to establish an independent dataset. The overall amount of the three activities (rest, walking, running) were 1697, 2320 and 2006 separately.

Table 8 shows the confusion matrix of the proposed solution for recognizing physical activities, in which each row shows how the model classified one class and each column shows which classes one type of classification by the model actually belongs to. The overall accuracy of 97.7% has demonstrated the performance of the proposed physical activity recognition approach. Therefore, the utilization of built-in-sensors in the smartphone for activity recognition for the proposed context-aware ECG system is feasible.

**Table 8:** Confusion matrix of J48 decision tree.

	Model	Walking	Running	Rest
Actual				
**Walking**		**2268**	51	1
**Running**		68	**1938**	0
**Rest**		16	0	**1681**

### 3.4. Performance of the Proposed Context-Aware ECG Monitoring System

The ECG signal is amplified and filtered by the chip of AFE module, then the analog signal from AFE is converted to digital signal in MCU module. After processed with wavelet processing algorithm and feature extraction comparison algorithms, the generated digital signal are recorded in the memory cells or transmitted to the personal phone for data fusion and analysis.

**Figure 9:**
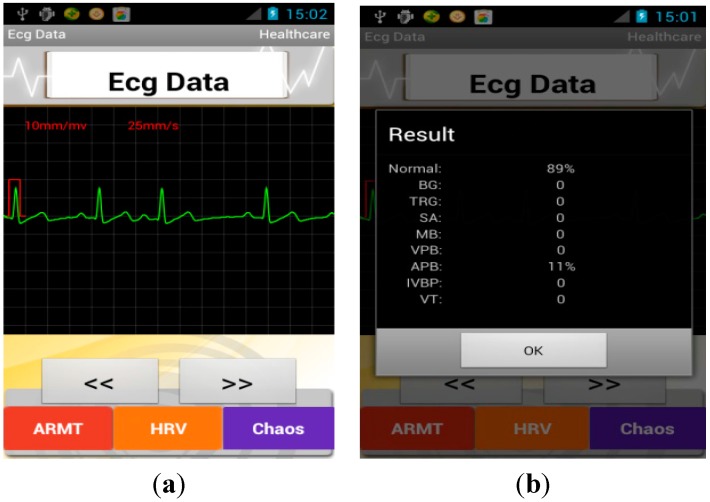
Screenshot of smartphone (**a**) abnormal ECG signal; (**b**) the brief report.

When the monitoring process completed, the features of subjects’ acquired ECG signal were extracted using WABS method and MLE method and according to these features a brief report aiming to detect 10-type arrhythmias and HRV analysis was given, as shown in Figure 9. The full names of the abbreviations are given in Table 9. The diagnostic capability was also validated using the muli-parameter simulator (MEDSIM 300B). The testbed is presented in appendix Figure 6, in which the multi-parameter simulator was used to generate ECG signals with different arrhythmias types. We evaluated the discrimination ability of in terms of half total error rate (HTER), which equals to (false acceptance rate (FAR) + false rejection rate (FRR))/2. The technical report is demonstrated in Table 10, from the Table 10 we can see, the software on smartphone can realize a good discrimination performance in recognizing abnormal pattern with high reliability.

**Table 9:** Abbreviations in the report.

Abbreviations	Full Name
BG	Bigeminy
TBG	Trigeminy
SA	Sinus arrhythmia
MB	Missed beat
VPB	Ventricular premature beats
APB	Atrial premature beats
IVBP	Interpolated ventricular premature beat
VT	Ventricular tachycardia
PB	Pause Beat

**Table 10:** Discrimination ability of the proposed software.

Items		True Positive	False Negative	True Negative	False Positive	Discrimination Ability (HTER)
**Arrhythmias Type**	BG	444	3	2024	0	0.34%
	TBG	258	0	2213	0	0
	SA	200	0	2271	0	0
	MB	125	1	2345	0	0.345%
	VPB	149	3	2315	4	1.04%
	APB	247	1	2220	3	0.27%
	PB	49	1	2421	0	1%
	VT	287	3	2179	0	0.5%
	Tachycardia	200	0	2271	0	0

**Figure 10:**
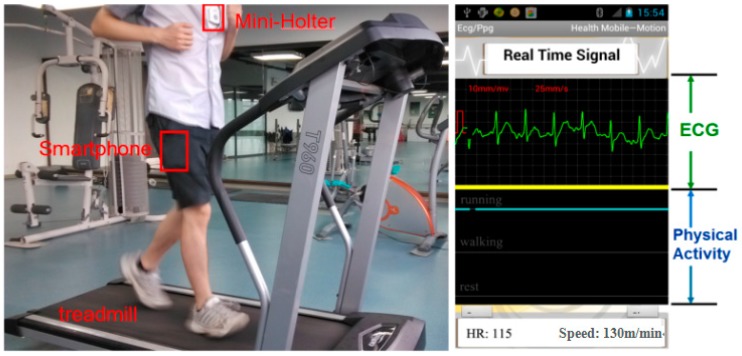
The experiment of ECG Acquisition system with physical activity recognition on a treadmill.

In the sensor fusion application, the subject wore the ECG acquisition sensor on the chest with the smartphone in any trousers pockets. Figure 10 gives a visible presentation of our experiment with one subject as the example. On the treadmill, the subject was running at the speed of 8 km/h. Moreover, the screenshot of the smartphone was also displayed in Figure 10. “HR” represents “Heart rate” in the figure, which indicates that the heart rate of the subject was 115 at this time. “Speed” represents the ambulation speed of the specific time duration, which can help to evaluate human efforts while performing the activity. The green line was the ECG signal at the real time, while the blue line indicated physical activity of the subject. When the blue line was located at the top, middle, bottom, it demonstrated that the subject was running, walking and resting at this moment, respectively.

**Figure 11:**
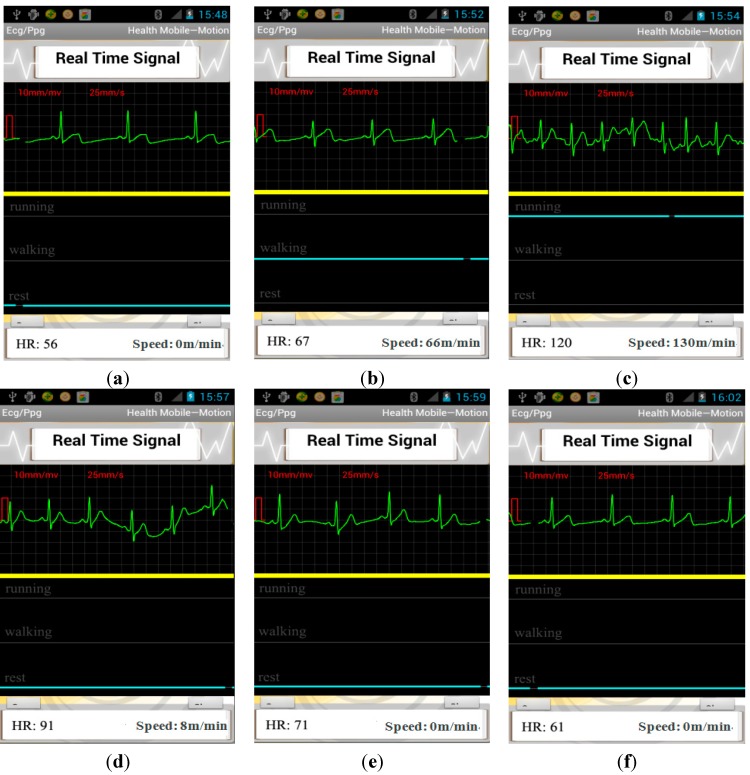
The screenshot of smartphone (**a**) the subject was resting; (**b**) the subject was walking on the treadmill; (**c**) the subject was running on the treadmill; (**d**) the subject rested 30 s after running; (**e**) the subject rested 90 s after running; (**f**) the subject rested 300 s after running.

A case for continuously monitoring one subject’s context-aware ECG with the proposed system is presented in Figure 11. From Figure 11a we can see, the subject is with a heart rate of 56 while resting. When the subject changed his activity status from walking to running, there was a sharp increase in heart rate of the subject from 67 to 120. Then, after running, the subject rested 30 s with a heart rate of 91. When the subject was resting 300 s after running, heart rate of the subject recovered to the value before running. From the above report we can see, the context-aware system is necessary to evaluate the user’s real health condition. For example, a heart rate of 120 is usually deemed as tachycardia from professional experience. However, it is acceptable if the user is running or taking exercise, which can be reflected from the activity recognition solution. In addition, the monitoring on the variation of ECG with the physical activity provides a useful tool to evaluate one’s cardiac function on the adaption to the change of status.

To demonstrate the usefulness of context-aware ECG to recognize physical activity while monitoring ECG, we also performed a statistical analysis on the improvement on the diagnosis accuracy combined with physical activity. Table 11 gives the abnormal patterns detected before and after combined with context information and the comparison with the actual patterns from the clinician’s diagnosis. The overall heartbeat number is 7100. VT is often classified as three or more beats on an ECG that are at a rate of more than 100 beats per minute in static status, however, when transferring from walking status to running status, the heart rate increased rapidly, perhaps in a range of 120–180. Therefore, VT can be defined as three or more beats are with a rate of more than 180 beats per minute in running status, as the volunteers are around 24 years old. In order to demonstrate the discrimination performance of the proposed context-aware ECG system, except for an intuitive presentation in Table 11, we also compared the HTER between the single ECG sensor and the context-aware ECG system on recognizing abnormal patterns, which can be seen in Table 12. From the table we can see, the discrimination ability was improved in a certain degree by combination of physical activity with a HTER of 2.6%, compared with 2.8% with the single ECG sensor, and thus demonstrated the effectiveness of the proposed context-aware ECG system in real-life application. In addition, the proposed system can identify the most frequent abnormal ECG patterns in different activities for each subject, and accordingly, we can provide helpful suggestions for him to be careful during this type of activity. For example, as our experiment is carried out on healthy subjects, the abnormal patterns have a very low rate; however, we also found that most abnormal patterns are occurring at running status. In another words, a lot of asymptomatic ECG patterns can be detected through monitoring context-aware ECG.

**Table 11:** Statistical analysis on the performance of proposed context-aware ECG system.

Abnormal Patterns	Detected from ECG Sensor	Detected from Context-Aware ECG Sensor	Actual
BG	0	0	0
TBG	0	0	0
SA	0	0	0
MB	0	0	0
PB	0	0	0
VPB	4	4 (3 in running, 1 in walking)	4
APB	5	5 (3 in running, 1 in walking, 1 in rest)	5
IVBP	0	0	0
VT	31	9 (8 in running, 1 in walking)	10
Tachycardia	6	0	0

**Table 12:** Discrimination ability comparison between the proposed context-aware ECG system and single ECG sensor. TP: true positive, FN: false negative, TN: true negative, FP: false positive.

	ECG Beat Number				Discrimination Ability (HTER)
	TP	FN	TN	FP	
**Single ECG Sensor**	18	1	7054	27	2.8%
**Context-Aware ECG System**	18	1	7081	0	2.6%

## 4. Conclusions

A wearable context-aware ECG monitoring system, which is comprised of a self-designed fully-integrated low-power ECG monitoring sensor and three built-in kinematic sensors of the smartphone for physical activity recognition and automatically arrhythmias detection, is presented in this paper. In the proposed system, a wearable ECG acquisition sensor with a total power dissipation of 12.5 mW is developed, and the whole sensor is very small with a size of 58 × 50 × 10 mm for wearable monitoring application. From the experimental results, the presented AFE and acquisition device can offer comparable performance to standard device on measuring ECG signal with less power consumption. Integrated with the built-in kinetic sensors of the smartphone, the system can recognize user’s physical activity with high accuracy and thus helps evaluate the real status which abnormal patterns of ECG at. The experimental results have also demonstrated its feasibility in improving accuracy for the diagnosis of arrhythmias. The proposed wearable and power-efficient ECG monitoring system is qualified for medical applications and will serve as a patient-friendly alternative option for continuous ECG monitoring. We anticipate it will become a more efficient platform for further data fusion and analysis.

However, the experimental data is still small with seven healthy subjects and three activities involved. In the future, we will dedicate to a comprehensive study on the impact of more activity types and human efforts on ECG signals from a large number of experiments based on the proposed system. Moreover, an updated ECG monitoring system will be further optimized with not only smaller size, lower weight, lower power consumption and higher CMRR, but also automatically identifying the abnormal patterns during daily activities. Further, a remote healthcare system with context-aware ECG monitoring will be developed with the help of a private cloud platform [34].

## Supplementary Files

Supplementary File 1
