# Supplementary material for: A Wearable Context-Aware ECG Monitoring System Integrated with Built-in Kinematic Sensors of the Smartphone
Source: Sensors (Basel). 2015 May 19;15(5):11465–84. doi: 10.3390/s150511465 (PMC4481936; doi:10.3390/s150511465)
Supplement: Supplementary File 1 [file sensors-15-11465-s001.pdf]

OPEN ACCESS

sensors

ISSN 1424-8220

www.mdpi.com/journal/sensors

Supplementary Information

A Wearable Context-Aware ECG Monitoring System Integrated with Built-in Kinematic Sensors of the Smartphone. *Sensors* 2015, *15*, 11465-11484

Fen Miao [1,2], Yayu Cheng [1], Yi He [3], Qingyun He [1,2] and Ye Li [1,*]

[1] Key Laboratory for Health Informatics of the Chinese Academy of Sciences (HICAS), Shenzhen Institutes of Advanced Technology, 1068 Xueyuan Boulevard, Shenzhen 518055, China; E-Mails: fen.miao@siat.ac.cn (F.M.); yy.cheng@blestech.com (Y.C.); qy.he@siat.ac.cn (Q.H.)

[2] Shenzhen College of Advanced Technology, University of Chinese Academy of Sciences, Shenzhen 518055, China

[3] High-field Magnetic Resonance Department, Max Planck Institute for Biological Cybernetics, Tuebingen 72076, Germany; E-Mail: yi.he@tuebingen.mpg.de

* Author to whom correspondence should be addressed; E-Mail: ye.li@siat.ac.cn; Tel.: +86-755-8639-2201; Fax: +86-755-8639-2299.

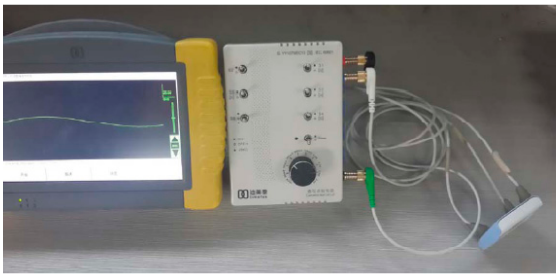

Figure S1. Testbed for input impedance.

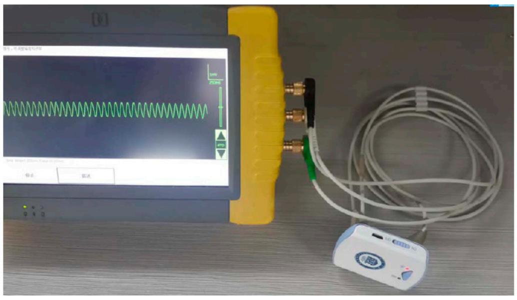

Figure S2. Testbed for frequency response.

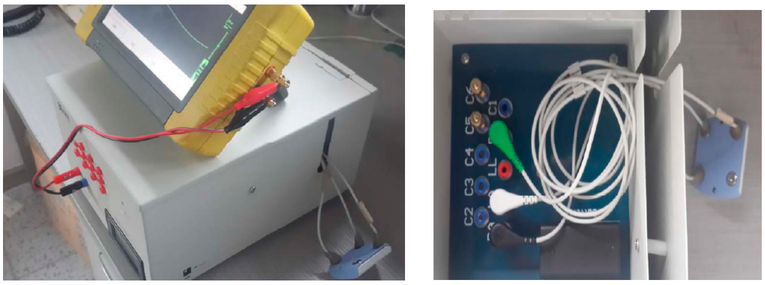

(a) **(b)**

Figure S3. Testbed for CMRR.

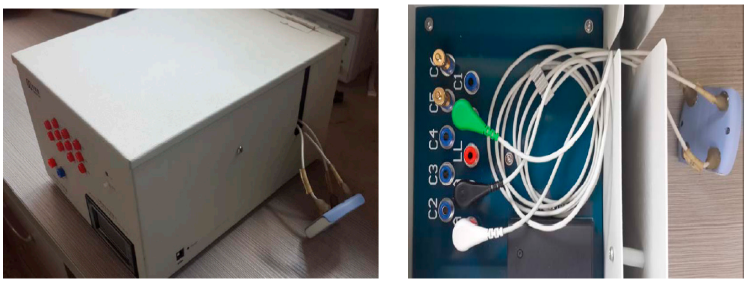

(a) **(b)**

Figure S4. Testbed for system noise.

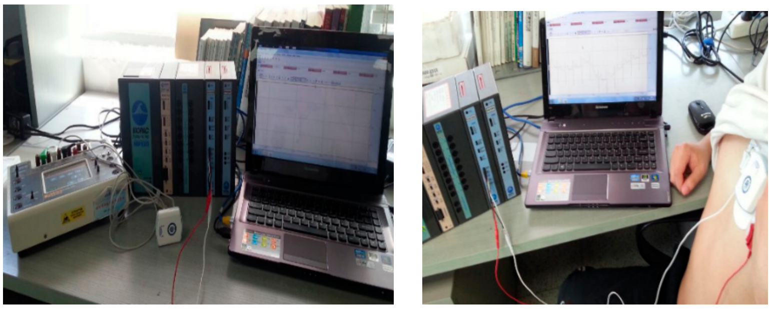

(a) **(b)**

Figure S5. Testbed for reliability of the proposed sensor on acquiring ECG signals.

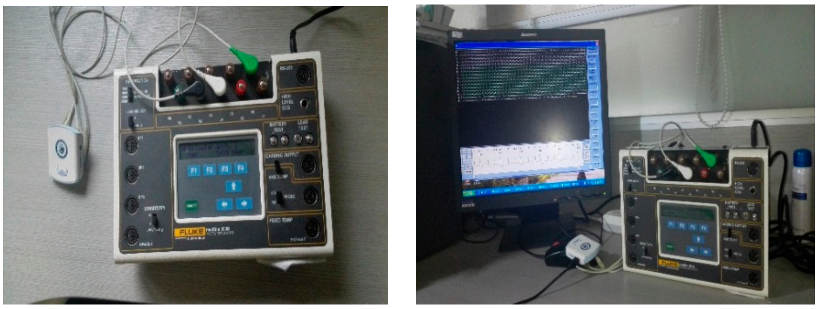

(a) **(b)**

Figure S6. Testbed for diagnostic capability.

© 2015 by the authors; licensee MDPI, Basel, Switzerland. This article is an open access article distributed under the terms and conditions of the Creative Commons Attribution license (http://creativecommons.org/licenses/by/4.0/).
